# Re-visiting the tympanic membrane vicinity as core body temperature measurement site

**DOI:** 10.1371/journal.pone.0174120

**Published:** 2017-04-17

**Authors:** Wui Keat Yeoh, Jason Kai Wei Lee, Hsueh Yee Lim, Chee Wee Gan, Wenyu Liang, Kok Kiong Tan

**Affiliations:** 1 Department of Electrical and Computer Engineering, National University of Singapore, Singapore, Singapore; 2 Department of Physiology, Yong Loo Lin School of Medicine, National University of Singapore, Singapore, Singapore; 3 Human Performance Laboratory, Combat Protection and Performance, DSO National Laboratories, Singapore, Singapore; 4 Department of Otolaryngology, Yong Loo Lin School of Medicine, National University of Singapore, Singapore, Singapore; St. Joseph’s Hospital and Medical Center, UNITED STATES

## Abstract

Core body temperature (CBT) is an important and commonly used indicator of human health and endurance performance. A rise in baseline CBT can be attributed to an onset of flu, infection or even thermoregulatory failure when it becomes excessive. Sites which have been used for measurement of CBT include the pulmonary artery, the esophagus, the rectum and the tympanic membrane. Among them, the tympanic membrane is an attractive measurement site for CBT due to its unobtrusive nature and ease of measurement facilitated, especially when continuous CBT measurements are needed for monitoring such as during military, occupational and sporting settings. However, to-date, there are still polarizing views on the suitability of tympanic membrane as a CBT site. This paper will revisit a number of key unresolved issues in the literature and also presents, for the first time, a benchmark of the middle ear temperature against temperature measurements from other sites. Results from experiments carried out on human and primate subjects will be presented to draw a fresh set of insights against the backdrop of hypotheses and controversies.

## Introduction

Core body temperature (CBT) is an important and commonly used indicator of the human health and endurance performance. The characteristics of CBT and the ideal measurement site have attracted extensive research in both clinical and the physiological domains. CBT is generated from the well-perfused tissues of the vital organs [1, 2] and thus, it remains relatively stable since heat distribution within these tissues is attained at a fast rate. As a result, the CBT of a healthy human body rarely differs and it is very close to the temperature of blood flowing in the pulmonary artery [3, 4]. Thus, the blood temperature of the pulmonary artery (*T*_*pa*_) is often taken as the gold standard in CBT measurement [3–6]. However, the measurement of *T*_*pa*_ is an invasive and high risk procedure, requiring the insertion of a pulmonary artery catheter. Thus, *T*_*pa*_ measurement is mainly restricted to critical care monitoring within a hospital environment [6], wherein cardiac or critically ill patients are being monitored round the clock.

Apart from the pulmonary artery, the esophagus and rectum are other sites to measure CBT [6–9]. The esophagus site requires rather invasive process to access, while the rectum site is rather inconvenient in term of its accessibility. These sites are inherently less risky compared to the pulmonary artery and certain considerations have to be observed in order to obtain accurate and consistent temperature measurements. Esophageal temperature (*T*_*es*_) is accurate when the probe is positioned at the lower fourth of the esophagus which is closer to the heart and aorta. If the probe is located too high up in the esophagus, the reading will be affected by tracheal air due to the breathing process [10, 11]. *T*_*es*_ can be a good candidate for the CBT due to its responsiveness which is on-par with *T*_*pa*_ [12], but due to the sensitivity to proper probe placement, the distress caused to patient during measurement and the associated cost and time, the esophagus is also not a common site for CBT.

Rectal Temperature (*T*_*rm*_) is considered as an indicator of deep tissue and critical tissue temperatures [10]. The large tissue mass surrounding the rectum provides for a stable temperature reading that is shielded from the surrounding environmental temperature [10, 13]. However, *T*_*rm*_ is not suitable as an indicator of CBT when there is a need to pick up rapid changes in the CBT [13, 14]. When the human’s body is at thermal equilibrium, *T*_*rm*_ is a reliable substitute of the CBT. But when the CBT is undergoing a transient period, *T*_*rm*_ is sluggish to pick it up as it exhibits slow response to changes in heat input and losses, and follows rather than leads the body’s thermoregulatory reactions [13, 14]. Hence, *T*_*rm*_ is limited in the ability to respond to thermal transients involving sudden or abrupt changes to CBT. For a critically ill patient, changes in CBT is crucial to determine the next treatment protocol [1, 3]. Such limitations have led to the suitability of *T*_*rm*_ to determine CBT being repeatedly questioned, though till now, the rectum is still a widely used CBT site due to the relatively efficient and stable measurements.

The aforementioned sites are not amenable towards general use or temperature monitoring in active personnel. Generally, all of them pose a certain amount of risk and discomfort (as well as embarrassment (e.g., *T*_*rm*_ measurement)) from the user perspectives and they can incur rather high costs. The ideal CBT site has, by far, remained elusive to-date. Thus, research continues to be undertaken to explore alternative temperature measurement sites which are conducive for general and continuous monitoring purposes, and are reliable sufficiently as an indicator of the CBT. One such site is the tympanic membrane. Tympanic membrane temperature (*T*_*tm*_) measurement via infrared thermometry has been touted as a potential replacement for the invasive temperature measurement procedures discussed. The safe, least invasive and arguably most comfortable way of measuring *T*_*tm*_, however, is not yet a de-facto standard to-date; not when there are still unresolved issues pertaining to its accuracy and stability relative to measurements from other sites [2, 5, 11, 15].

Benzinger [16–18] first demonstrated the feasibility of *T*_*tm*_ measurement as an indicator of CBT using a thermocouple temperature probe engaging the surface of a tympanic membrane with the ear canal sealed off from the environment. Benzinger hypothesized that the tympanic membrane, being in close proximity to the hypothalamus and to the internal carotid artery (and since the internal carotid arterial blood flow perfuses the tympanic membrane), is most suitable for accurate indication of CBT. To support his hypothesis, Benzinger produced measurements of *T*_*tm*_ obtained from his probe and measurement approach, showed them to be stable, reproducible and responsive to thermal stresses of various kinds, and also demonstrated *T*_*tm*_ to be more responsive than *T*_*rm*_ in indicating CBT.

On the other hand, subsequent studies by McCaffrey *et al* [19] and Nielsen [20] indicated that head cooling, particularly when the cooling is directed at the facial area, decreases *T*_*tm*_. McCaffrey *et al* showed that by heating and cooling localized regions of the head, *T*_*tm*_ of human subjects were non-proportionally affected by changes in the skin temperature of the head. These results meant *T*_*tm*_, by itself, is not a good indication of CBT since it is affected by other variables.

**Fig 1:**
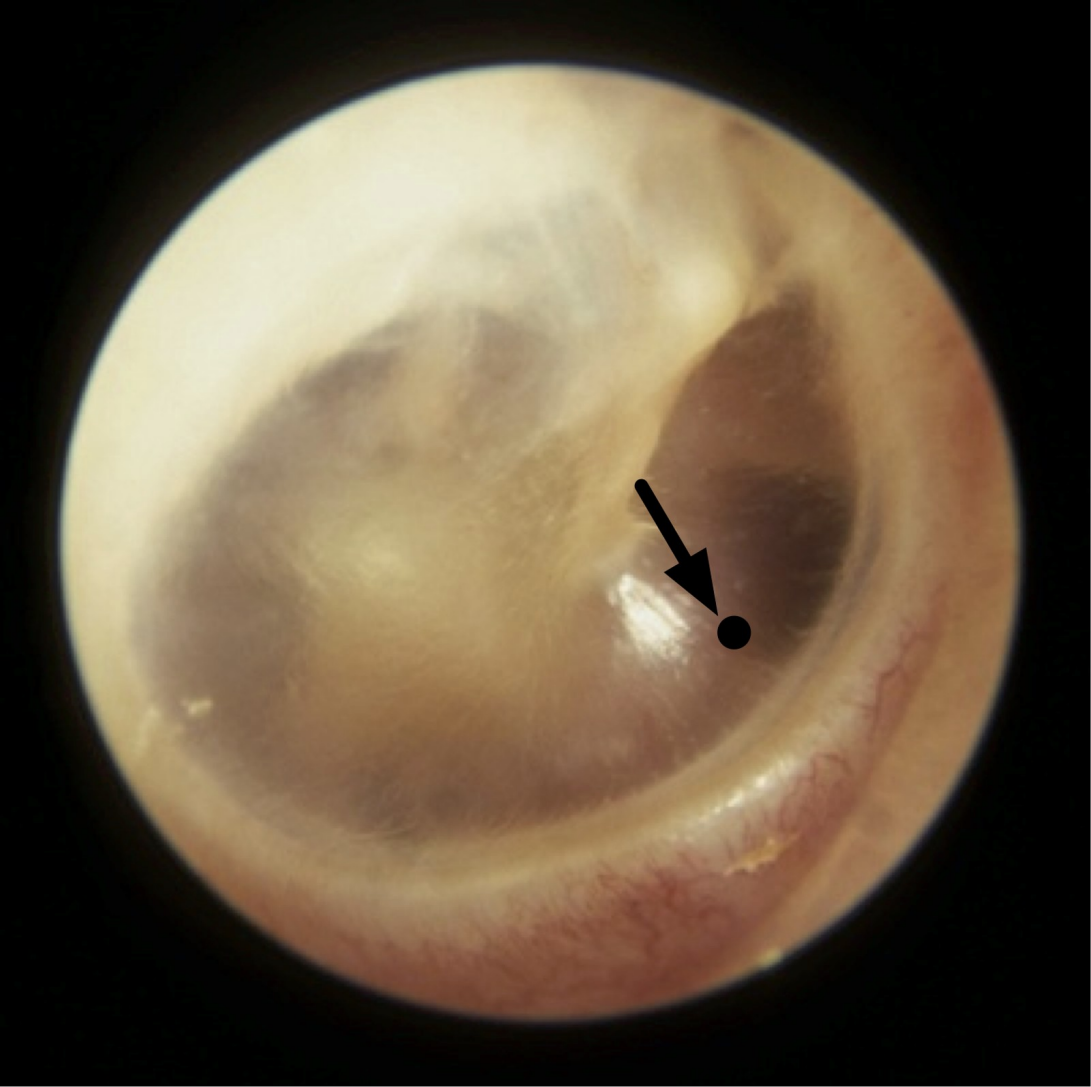
Measurement spot on the tympanic membrane. Tympanic membrane of the right ear showing a temperature measurement spot at the lower anterior quarter (pointed by the arrow).

However, Brinnel and Cabanac [21], and Sato *et al* [22] furnished data to show that *T*_*tm*_ is still reliable for this purpose if the measurement spot on the tympanic membrane is chosen with care and an adequately directed probe is used to acquire the measurements. Brinnel and Cabanac suggested that the lower anterior quarter of the tympanic membrane (Fig 1) has a higher temperature on the surface of the tympanic membrane and a temperature measurement from a point in this region is least sensitive to head cooling. Sato *et al* performed similar experiments as Brinnel and Cabanac, and they determined the highest measured temperature spot on the tympanic membrane with a trial and error approach of probe positioning until the hotspot is found. They produced results, with the probe engaging this spot on the tympanic membrane, showing the measurements obtained were least sensitive to the effects of head cooling. However, they did not define the point of measurement.

These works were done with the probe engaging the surface of the tympanic membrane which is neither practical nor safe from perforation of the tympanic membrane when taking measurements from awake patients, especially when measurements are required continually. However, the need to engage the tympanic membrane can be understood since the surface of the tympanic membrane is not flat but conically shaped and a physical contact is essential to ensure that the measurements from all sites on the surface can be compared in a fair manner. The ideal spot which can be easily located for measurement has also not been clearly specified and substantiated by previous studies.

This paper was motivated by the above-mentioned issues and gaps in allowing *T*_*tm*_ to be used as an inference to CBT. In addition, the paper sought to explore, to the best of our knowledge for the first time, an alternate CBT measurement site in the middle ear cavity. Being located at the inner side of the tympanic membrane, we aimed to shed light on the existing gaps in the use of the ear as a CBT site. The middle ear cavity resembles a rectangular chamber with four walls, a ceiling and a floor. The lateral wall being the tympanic membrane, the ceiling is a thin plate of bony structure that separates the chamber from the cranial cavity and the brain, and the floor is another thin bony plate that separates the chamber from the jugular vein and the carotid artery. The posterior wall partly separates the chamber from the mastoid antrum. The anterior wall is the opening of the eustachian tube, which connects the chamber with the nasopharynx. The medial wall separates the chamber from the inner ear [23]. This site selection is appropriate since the middle ear cavity is very near to the brain (ceiling), jugular vein and carotid artery (floor), and it is surrounded by highly perfused tissues. The temperature at this cavity (*T*_*me*_) can potentially reflects a stable temperature to infer CBT. The reason for the lack of effort in the exploration of this site may be attributed to the difficulty in the access to the middle ear. However, in recent years, advances in medical surgery have led to the simplification of the procedure to carry out a ventilation tube insertion on the tympanic membrane to an office-based procedure [24, 25] without any need for general anesthesia. A channel can thus be readily created to access the middle ear via the tube for temperature measurements. An alternate access path is also possible through the Eustachian tube [26].

Specifically, we aimed to: (1) examine the surface temperature distribution at a small but within constant proximity from the tympanic membrane, and the identification of a clear landmark for the measurement of *T*_*tm*_. The results can be potentially useful in the design of tympanic membrane thermometers to infer close readings to CBT in the face of environmental cooling. (2) benchmark the middle ear temperature against current CBT sites (including the tympanic membrane), with a side objective to observe if *T*_*me*_ shows characteristics which can be observed from *T*_*tm*_. The results can potentially be useful towards further exploration of the middle ear as a CBT site. Apart from these two issues, we verified the cooling effects on the measurements of *T*_*tm*_, the relative responsiveness and repeatability of measurements in *T*_*es*_ and *T*_*rm*_, and the correlation among temperatures from the various sites.

We applied for the necessary approval from the SingHealth Institutional Animal Care and Use Committee (IACUC) prior to the commencement of the experiments involving a live animal subject. The IACUC reviewed the experiments procedures and concluded that the experiments were proper and humane, hence we were given the approval (REF:2012/SHS/712) to proceed with the experiments as planned.

The paper is organized as follows. In Exploration Focal Points, we will lay down the two main issues to be explored in the paper, the background behind them and the potential outcomes from the investigations set forth. In the Design of Experiment section, we will highlight the experiments and procedures, subjects and instruments used. In the Results and Discussions section, the results from the experiments will be presented and discussed. Finally, conclusions will be drawn in the last section.

## Exploration focal points

It should be acknowledged at the outset that the study of CBT is an extensive and challenging one. It is not realistic to expect an exhaustive and comprehensive set of results from any single and specific work due to the complexity and the nature of the human body and the constituent organs, as well as the dynamic interactions with endogenous and exogenous influence. The paper seeks to focus on two specific issues (1. Temperature Uniformity in the Tympanic Membrane Vicinity, and 2. Middle Ear Cavity as CBT Site) to revisit the tympanic membrane vicinity as a potential CBT site. These two issues will be clearly formulated with respect to the background work and gaps, and the exploration approaches projected to align with the experiments and results which will be presented in the ensuing subsections.

### Temperature uniformity in the tympanic membrane vicinity and measurement point selection

While the outer ear is an excellent site for temperature measurements, it is well-known that the readings from commercial ear thermometers are lacking in repeatability and accuracy [2, 5, 11, 15]. One main reason for this phenomenon is the different ear anatomy from one person to the next, and thus the difficulty in the design of a general-purpose probe which can be inserted into the outer ear with good sealing from the environment and with the probe distal end well directed at a desirable measurement point. This has prompted us to first investigate on the ideal measurement point or region within the ear. It is important to orientate the probe to a specific ear anatomy. Previous studies [19, 20] have shown that arbitrary measurement points on the tympanic membrane can lead to measurements that are very sensitive to external effects such as face cooling.

Brinnel and Cabanac [21], and Sato *et al* [22] proposed probes engaging the tympanic membrane surface at restricted points on the tympanic membrane, but the exact location of these points were not clear nor in concurrence. Brinnel and Cabanac suggested a measurement spot at the lower anterior quarter of the tympanic membrane would have the highest temperature and is least sensitive to head or face cooling. Sato *et al* also sought to find the hottest spot on the tympanic membrane with trial and error by positioning the probe until the hotspot was found. The probe was engaged to the tympanic membrane at all the measurement points so that the results can be interpreted on a level field with respect to the convective heat losses which will be minimized with contact. This common requirement to physically engage the tympanic membrane is reasonable and sound since the tympanic membrane is not flat but conically shaped. Engaging the tympanic membrane is a way to ensure all temperatures are taken at the same proximity to the tympanic membrane in order to fairly select the hottest spot. However, in practice, it is unrealistic and unsafe to take *T*_*tm*_ measurement this way, with the probe engaging the tympanic membrane in a conscious individual.

The selection of the hottest spot is a common recommendation from the above mentioned studies [21, 22], though Sato *et al* did not put a boundary to the spot while Brinnel and Cabanac suggested the lower anterior quarter shown in Fig 1. Choosing the spot with the highest temperature is reasonable too since this is likely to be the point where it is most stable against environmental influence. However, it is noteworthy that these experiments were conducted in settings wherein the environment temperature was lower than the CBT. Should the opposite happen, heat flow would change direction and we could be seeking the coolest point instead when the body seeks to thermoregulate and maintain CBT against external heat stress.

**Fig 2:**
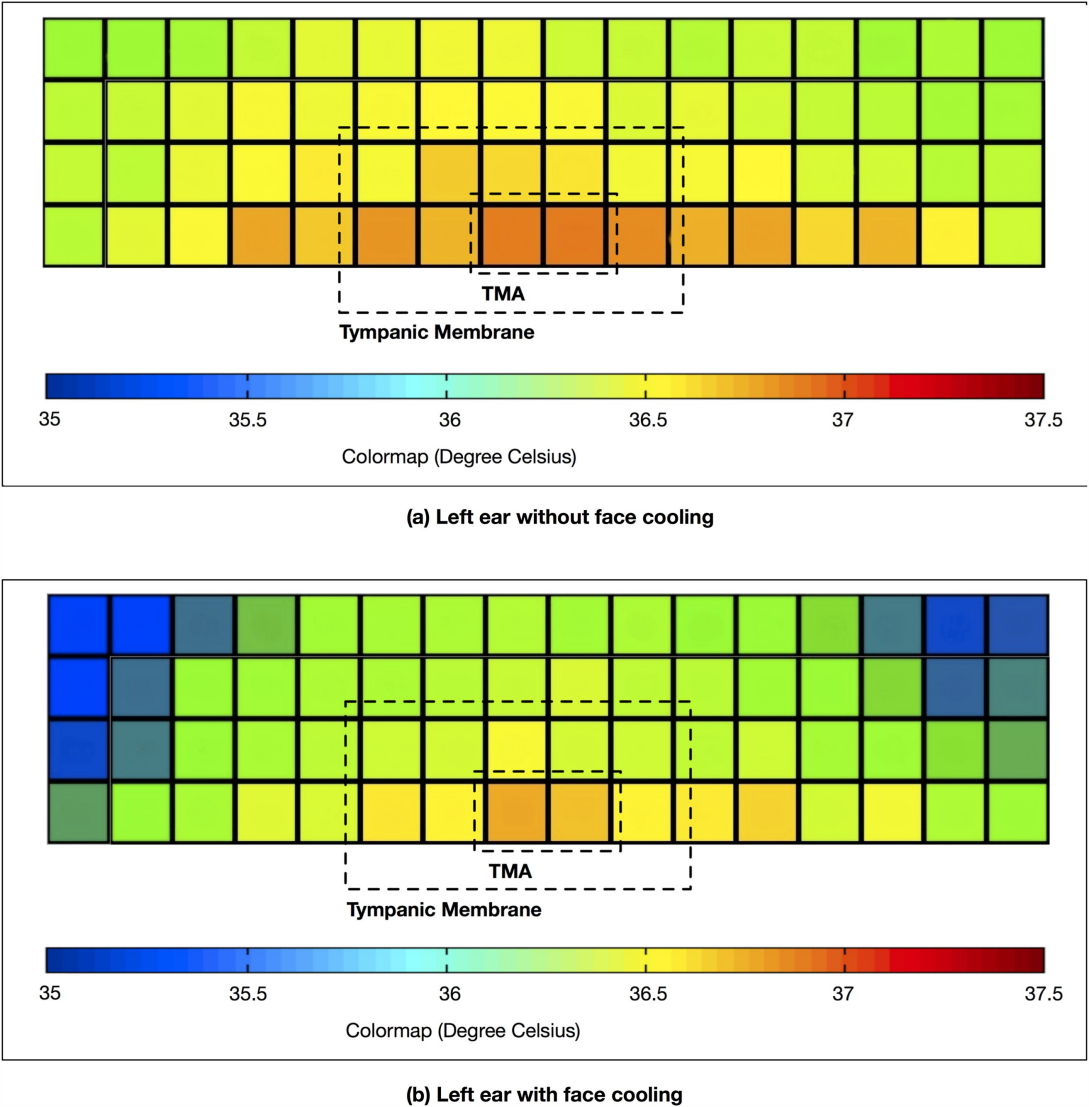
Temperature uniformity maps. Temperature uniformity maps of the left ear: (a) without face cooling and (b) without face cooling.

Preliminary tests and simulation were done to explore the uniformity of temperature distribution on the tympanic membrane. A non-contact thermopile sensors array (TSA) was used to measure the surface temperature of the tympanic membrane of a healthy volunteer. A speculum of an appropriate size was first inserted into the ear canal to straighten it. The TSA was positioned within the speculum to measure the temperature of the exposed tympanic membrane. The Melexis MLX90620 TSA was used which contains 64 infrared pixels arranged in a 16 × 4 rectangle area with an optical field of view of 60° to detect far infrared (FIR) induced thermal radiation. It can measure temperatures without making direct contact with the tympanic membrane. Each pixel has a 16-bit temperature measurement resolution, and all pixels have been factory calibrated with an accuracy of ±1.5°*C* for temperature measurements within the range of 0 to 50°*C*. Fig 2(a) depicts the measurement results showing the flattened temperature uniformity map of the ear canal space wherein the dotted line rectangle contains the temperature measurements of the tympanic membrane. Here, our interest is to show up the temperature deviation across the tympanic membrane and the canal area. Thus, different shades of color were used represent the relative temperatures. Shades of blue represents the lowest temperature, followed by green, yellow and red (highest temperature). In this measurement, due to the anatomical constraint, only half of the tympanic membrane was exposed to the direct line of sight of the TSA. The measured temperatures are non-uniform across the view window including the tympanic membrane. The measurement results in Fig 2(b) show the effects of face cooling. In general, with the cooling, it can be observed that the tympanic membrane temperatures as well as those at the surrounding ear canal walls lowered in response to the cooling, though not at the same proportion. The region with the highest temperature is observed (and marked as “TMA”) within the demarcated tympanic membrane area. The “TMA” area in Fig 2(b) is observed to be less sensitive to the effects of face cooling.

**Fig 3:**
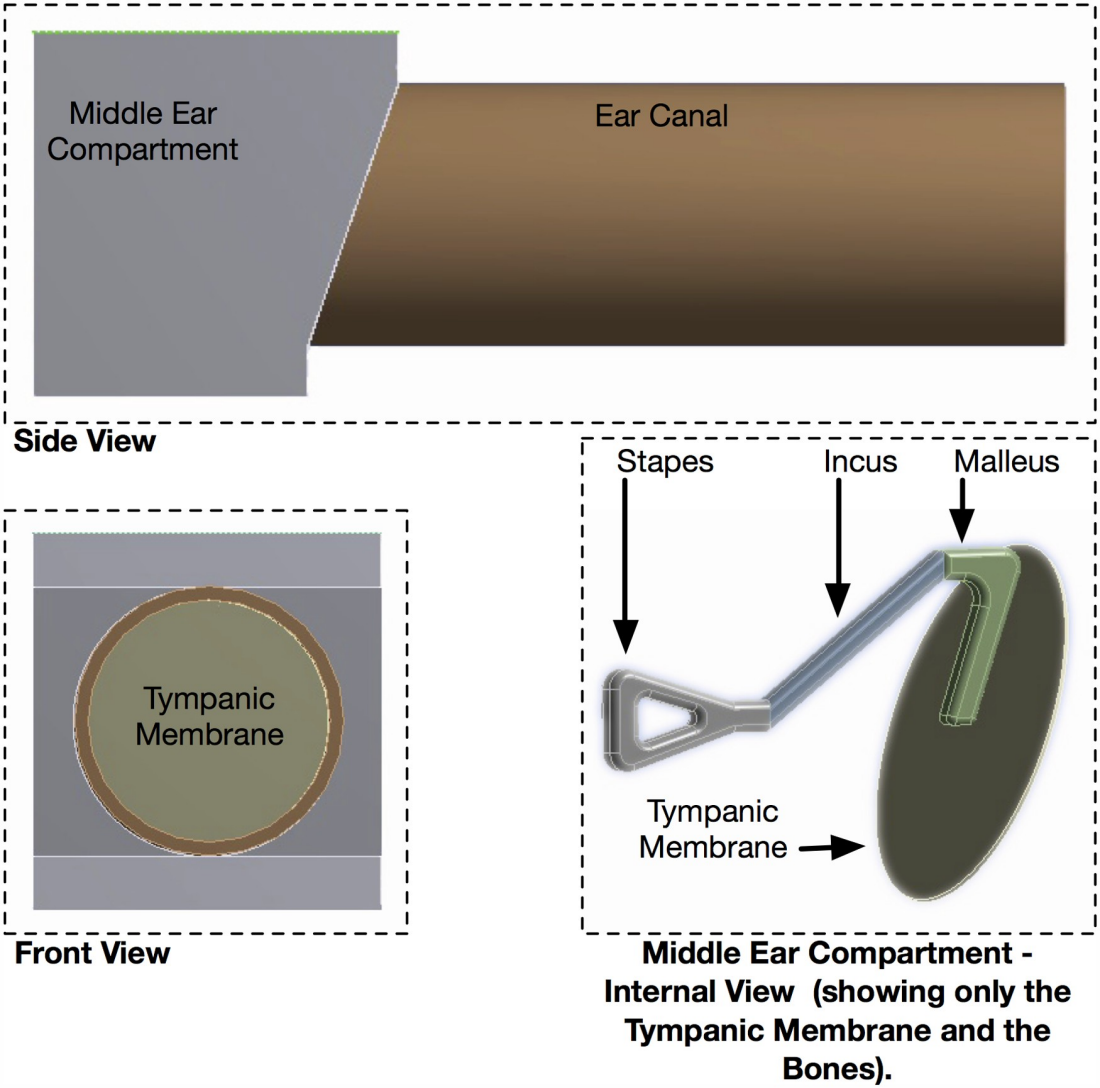
Human ear model. Human Ear Model used in Finite Element Analysis.

Finite Element Analysis (FEA) simulation was performed on a model of a human ear canal and middle ear compartment (Fig 3). The tissues and bones parameters were selected to closely match those of reported human tissues and bones at these two sites. The purpose of the FEA is to verify the temperature non-uniformity on the tympanic membrane surface and at 1mm above the surface. The tympanic membrane was modeled as a circular disc of 9mm in diameter and 0.1mm thick with malleus, incus and stapes bones attached from behind, and the front attached to the ear canal. The ear canal was modeled as an air filled hollow cylinder of 9mm in diameter and 25mm in length. The middle ear was modeled as a rectangular compartment with six walls, and the tympanic membrane was attached to the lateral wall. It is assumed that the superior wall is near to the brain, the inferior wall near to the carotid artery supplies the main heat source to the middle ear compartment and the perfused tissues surrounding the middle ear and the ear canal serve as heat sources. In the FEA simulation, the initial steady state temperatures of the malleus, incus and stapes bones location within the enclosed middle ear were correlated to the heat emitted from the brain, the carotid artery and the perfused tissues.

**Fig 4:**
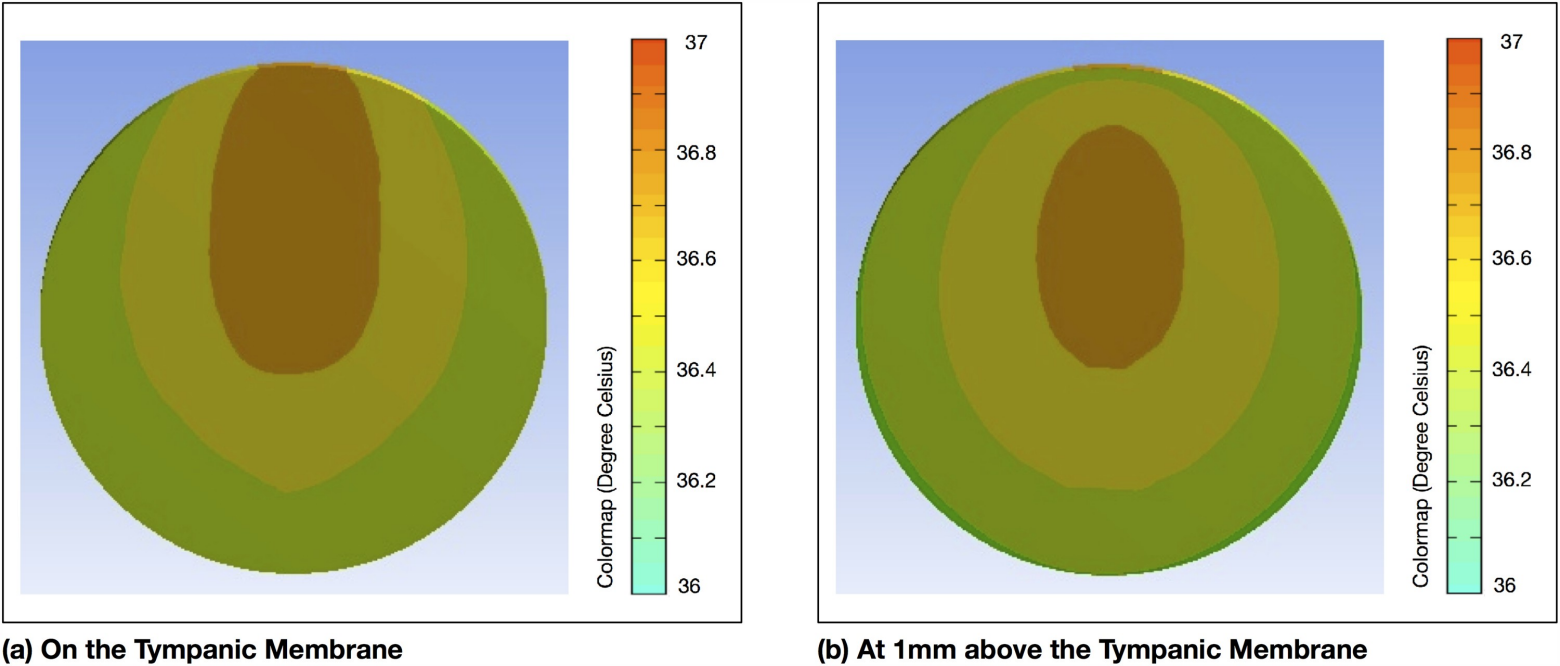
FEA results. FEA showing temperature uniformity: (a) on the tympanic membrane and (b) at 1mm above the tympanic membrane.

The tympanic membrane temperature uniformity map of the tympanic membrane surface is shown in Fig 4(a). From this simulation, we observe the area near to the malleus bone registered the highest temperature. This may indicate that the point of maximum temperature may not necessarily always be sited in the lower anterior quadrant [21] and depending on individual ear anatomy may lie elsewhere. The temperature uniformity map at 1mm above the tympanic membrane surface is depicted in Fig 4(b). Here, while the temperatures may be lower generally due to convective losses, the uniformity has remained rather consistent with the that at the tympanic membrane surface (Fig 4(a)). It may not be necessary to physically engage the tympanic membrane for the purpose of locating the highest temperature point.

**Fig 5:**
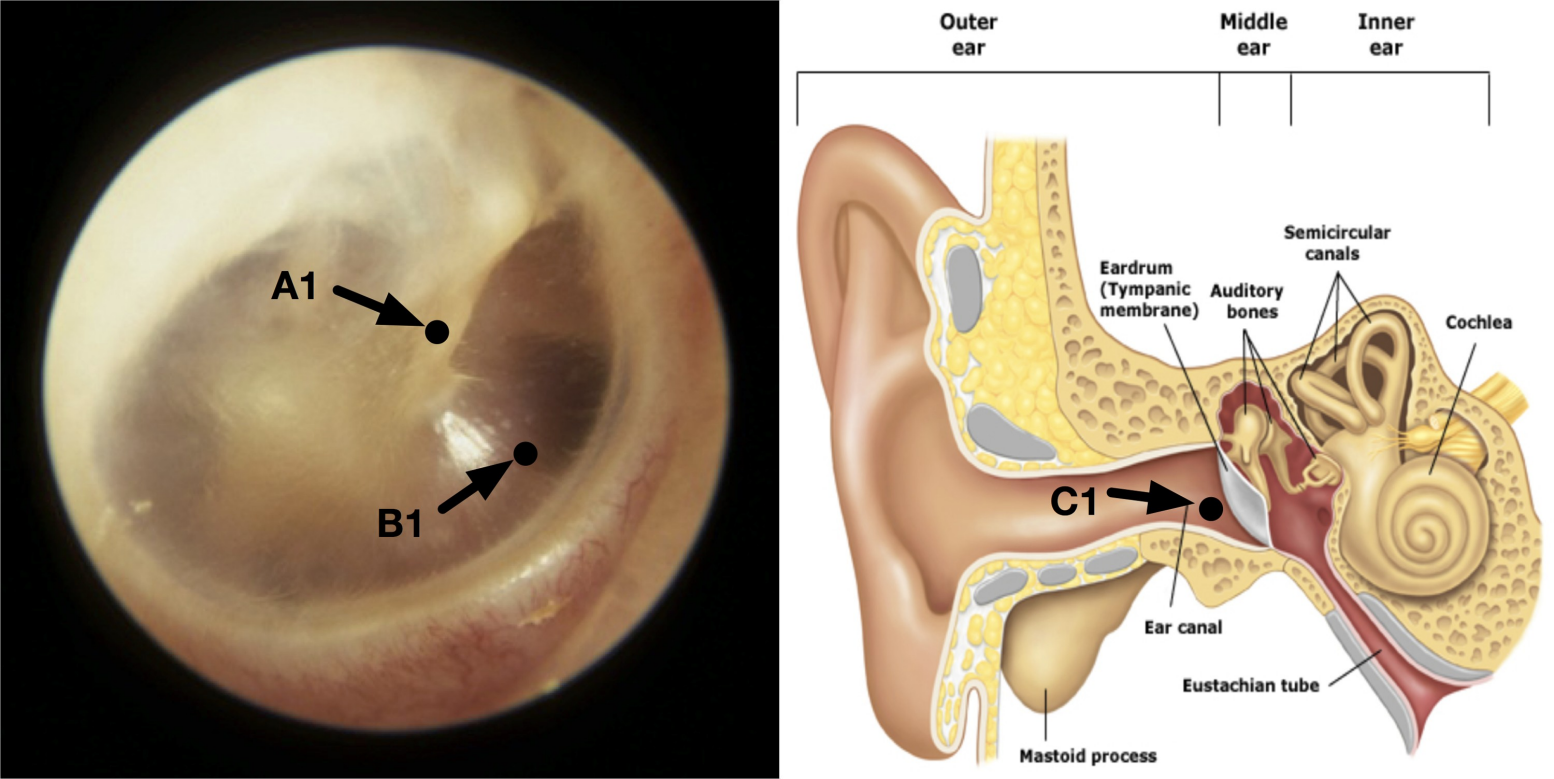
Temperature measurement points for Experiment 1. Tympanic membrane of the right ear with temperature measuring points labeled as A, B and C.

With these observations, we next aimed to experimentally obtain temperatures at accessible and potential points on the tympanic membrane without a physical engagement of the tympanic membrane, but maintaining the same small but uniform proximity from probe to the tympanic membrane, using a modified probe capable of proximity sensing. We would verify the temperature uniformity of these points on the tympanic membrane, and from the results, select the most appropriate point for measurement. As potential candidates, we chose a point in front of the malleus bone (Point A), a point in the lower anterior quarter (Point B) and a point at the distal end of the ear canal nearest to the tympanic membrane (Point C) (Fig 5). Point A was chosen because the tissue near to the malleus is thicker and accordingly a likelihood of a higher concentration of blood vessels [27]. Our conjecture, following the FEA analysis, is that this point has a larger thermal mass than those further away from the malleus bone. Point B is at the lower anterior quarter of the tympanic membrane. It was selected as suggested by Brinnel and Cabanac [21]. Point C lies on the canal just adjoining to the tympanic membrane. It was selected to serve as a comparison to A and B measurements, and it would represent the point at which temperature is typically taken with a wrongly directed ear thermometer.

Experiment 1 was conducted to enable this investigation. In Experiment 2 on a sedated primate, we verified the effects of facial cooling on *T*_*tm*_ by measuring *T*_*tm*_ in both ears but fanning only one side of the face. The effects on other CBT sites, including the esophagus and rectum, would also be studied.

The outcomes from this investigation will be beneficial in the design of ear thermometers to infer a temperature that is most representative of CBT.

### Middle ear cavity as CBT site

As explained in the Introduction, the middle ear cavity is closest to the brain than the other CBT sites. It holds unexplored potential to be an usable CBT measurement site. Till recently, accessing the middle ear space is challenging and it is not a natural approach for the purpose of taking a temperature reading. However, with the advent in technology, it is now a simple office-based procedure [24, 25] to incise a small opening on the tympanic membrane and to maintain a small opening with a ventilation tube. An alternate access to the middle ear space is via the Eustachian tube which can be facilitated too in an office setting.

An experiment (Experiment 3) on a sedated primate was completed to log temperatures from the middle ear and other CBT sites (esophagus, rectum) under passive hyperthermia. In addition, it is known that the administration of general anesthesia will lower CBT [1, 9]. An interesting time window, when general anesthesia is removed during the experiment, is available for scrutiny into the relative responsiveness of the CBT candidates following a change in CBT due to the influence of general anesthesia.

From the results of these tests in Experiment 3, we compared the temperature measured at two different points on the tympanic membrane against the temperature measured from the middle ear (presumed as the CBT benchmark) to show the sensitivity of the *T*_*tm*_ measurements with respect to locations.

## Design of experiments

The setup of the experiments and the procedures prescribed are highlighted to provide the background in the interpretation of the experiments and results subsequently.

### Equipment

The key equipment used in these experiments include the temperature probes and the custom heating chamber. The details of these equipment are given in the ensuing subsections.

#### Temperature probes

Clinical temperature probes (Tympanic Membrane: D-TM1A, Esophagus: D-OS4A, Rectum: D-RB3A and Middle Ear: D-N1205) supplied by Exacon Scientific A/S were used in the experiments. The thermistor-based probes for measuring *T*_*tm*_, *T*_*me*_, *T*_*es*_ and *T*_*rm*_ have a consistent measurement accuracy of ±0.1°*C* for a temperature range between 25 and 50°*C*. The National Instruments Data Acquisition (NI-DAQ) system was used with a custom-built temperature recording software for automated temperature data acquisition from the attached temperature probes. The sampling interval was set to 0.1 second. The probes were first calibrated by immersing them in a glass container with 500ml of distill water at an initial temperature of 30°*C*. The glass container was placed in a heating chamber with the interior air temperature set to 65°*C*. The water was slowly heated within the chamber until it reached the target temperature of 50°*C*. The variations in temperature were logged for each of the probes. Using the temperature profile of the *T*_*rm*_ probe as a reference, the calibration curves were derived for each of the temperature probes to ensure they were equalized to give homogeneous response in the experiments.

**Fig 6:**
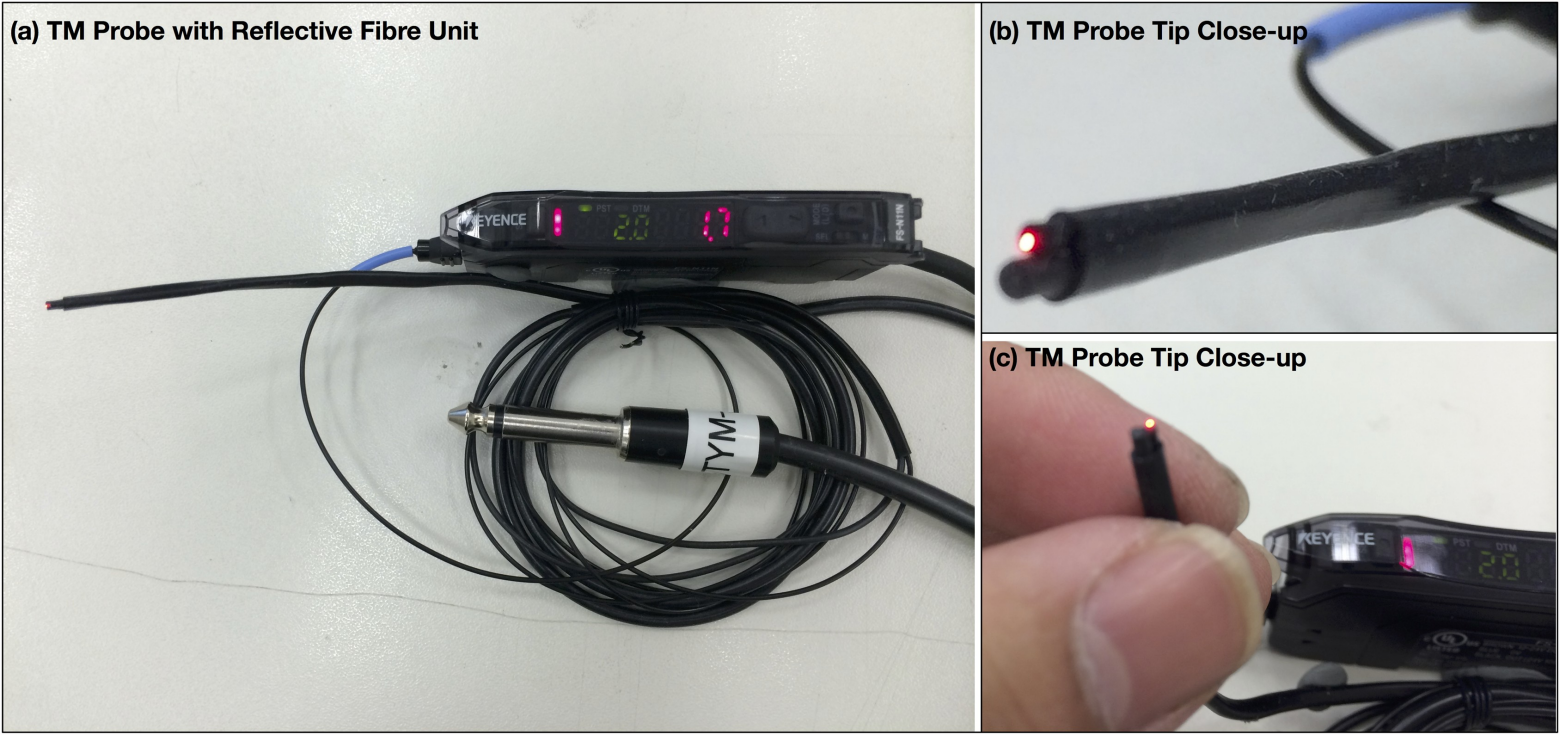
Modified clinical tympanic membrane probe. Clinical tympanic membrane probe supplied by Exacon Scientific A/S retrofitted with a Keyence Reflective Fibre Unit for use in Experiment 1.

In Experiment 1, it was a deliberate intent that, unlike previous work, the probe would not physically engage the tympanic membrane at these points, but at a very small but consistent proximity to them to observe the temperature difference between them in a way more typical of how a tympanic membrane temperature is taken. Modification to the *T*_*tm*_ probe was done to achieve this. It was retrofitted with a Keyence Reflective Fibre Unit (Model: FU-46 attached to a matching Fibre Amplifier Model: FS-N11N) at 1mm behind the sensing tip (Fig 6). This allowed the handler of the probe to be prompted when the probe was brought to a distance of <1mm from the defined measurement point for temperature measurement.

#### Heat chamber and probes setup

**Fig 7:**
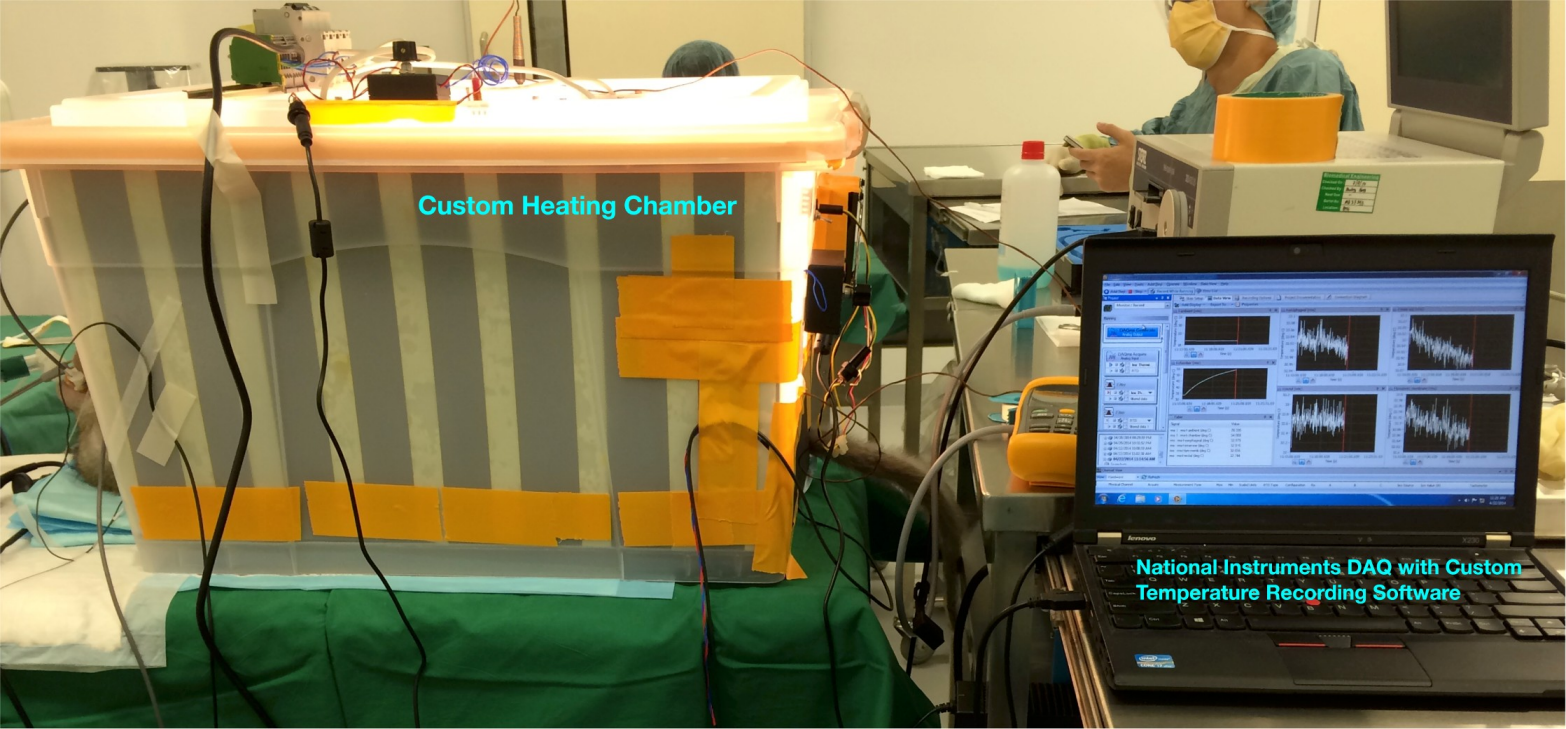
Custom heating chamber. Custom heating chamber constructed for use in Experiments 2–3.

A custom-built heat chamber similar to a small size incubator was designed and fabricated for the purpose of elevating the primate’s body temperature (full body passive hyperthermia) under controlled environment (Fig 7) in Experiments 2 and 3. The primate was sedated with general anesthesia in the chamber with the body from the neck downwards fully enclosed within the chamber. Only the head was exposed to external ambient temperature (*T*_*am*_) of the clinic. The trapped air in the interior of the chamber was heated with a 300W electric filament heater to reach a chamber temperature (*T*_*ch*_ = 65°*C*). Fans, controlled by an electronic thermostat, were used to circulate the heated air in-order to ensure temperature was distributed evenly *T*_*ch*_ ± 2°*C* within the chamber. *T*_*ch*_ was maintained until the primate’s esophageal or rectal temperature reached 42°*C* (the limit approved by IACUC), after which the heater was turned off before the chamber was opened to begin the cool-down process. Temperature probes were inserted into the primate’s body at defined measurement locations to measure the temperatures at these sites. Additional probes were used to measure *T*_*ch*_ and *T*_*am*_. Measurements were sampled every 0.1 second interval at each of the temperature probes. The raw temperature data sampled from the temperature probes were processed to remove noises and calibrated to ensure uniform responses.

### Procedures

The procedures behind the three controlled experiments are elaborated in the ensuing subsections.

#### Experiment 1

In Experiment 1, temperature measurements using the probe (retrofitted with proximity sensing) were taken at the defined points (A, B and C) on the tympanic membrane surface of a healthy adult human volunteer.

The ear canal has a diameter of about 5–8 mm in adults. Even though the modified probe has an outer diameter of 2.3mm, there is limited manoeuvring space to accurately access more points and not cause distress to the volunteer. The three points defined earlier in the Exploration Focal Points section were located for measurements; Point A in the vicinity of the malleus bone, Point B in the lower anterior quarter, and Point C at the distal end of the ear canal closest to the tympanic membrane as shown in Fig 5.

Under the view of a surgical microscope in a clinical setting with an ambient temperature of 22.5°*C*, an otolaryngologist guided the modified probe to the three points. The proximity sensor provided an alert when the probe reached a preset value of 1mm from the tympanic membrane. The same proximity was observed for measurements at all the three points. Steady state temperatures were logged after the measurements have stabilized.

#### Experiment 2

Experiment 2 was conducted to observe the effects of facial cooling on *T*_*tm*_, *T*_*es*_ and *T*_*rm*_ measurements. Probes were inserted by a veterinarian into a sedated primate to acquire real-time temperatures at these sites when it is placed within the heat chamber set to a temperature of *T*_*ch*_ = 65°*C*. At the mid-point during the passive hyperthermia process, convection skin cooling was initiated at the head area near to the left ear (left face fanning) by blowing cold air at *T*_*am*_ = 20°*C* with a small electric fan. The head area near to the right ear was shielded from the cooling process. Four temperature probes were used throughout the experiment: (a) Left tympanic membrane probe touching the left tympanic membrane’s surface for measuring *T*_*tm*,*L*_, (b) Right tympanic membrane probe touching the right tympanic membrane’s surface for measuring *T*_*tm*,*R*_, (c) Rectal probe was inserted via the anal sphincter for measuring *T*_*rm*_, and (d) Esophageal probe was inserted into the esophagus for measuring *T*_*es*_.

#### Experiment 3

In Experiment 3, the temperature of middle ear cavity was measured, for the first time, on its suitability as an inference to CBT. There were two parts to this experiment: Experiment 3*A* and Experiment 3*B*. In Experiment 3*A*, *T*_*me*_ was benchmarked against *T*_*tm*_ and *T*_*rm*_ with the sedated primate undergoing full body hyperthermia within the chamber set to *T*_*ch*_ = 65°*C*. Note that Experiment 2 and Experiment 3 were carried out on the same primate but the trials were separated by two months in order to allow sufficient time for primate to recover. Three temperature probes were used: (a) A needle probe to perforate the left tympanic membrane to reach the middle ear cavity just in front of the malleus bone for measuring *T*_*me*_, (b) Tympanic membrane probe engaging the right tympanic membrane’s surface to measure *T*_*tm*_, and (c) rectal probe inserted via the anal sphincter to measuring *T*_*rm*_.

Experiment 3*B* presented an ideal opportunity in the experiment setting to trigger a change in the CBT and to observe the relative measurements at various sites including the middle ear. The CBT trigger (approved by IACUC) was achieved by halting the administration of general anesthesia for 15 minutes, 2 hours and 51 minutes into the experiment. An additional probe was inserted into the primate’s esophagus to measure *T*_*es*_. Thus, a total of four temperature probes were used to each measure *T*_*me*_, *T*_*tm*_, *T*_*es*_ and *T*_*rm*_ throughout this experiment.

Two different spots were selected on the tympanic membrane for the measurements of *T*_*tm*_ in Experiments 3*A* and 3*B*. By comparing with *T*_*me*_, the sensitivity of the measurement points on *T*_*tm*_ could also be observed.

### Ethics consideration

Experiment 1 required non-contact temperature measurements from the surfaces of the ear canal and tympanic membrane of a healthy adult human. This procedure was conducted on one of the researcher who also co-authored this paper. The researcher has volunteered and consented to be part of the experiment. Approval from the Institutional Review Board (IRB) was not sought or required as the volunteer is a co-author (with no conflict of interest) and no other volunteers who were not part of the research were engaged for the experiment. The temperature measurements were performed without any physical contacts to the volunteer’s body surface by an Otolaryngologist (co-author), with no physical risk of ear injury.

A live adult primate (breed: Cynomolgus monkey, age: 6 years, weight: 5kg) was engaged for two rounds of experiments involving: (a) elevation of primate body temperature up to 42°C, (b) measurement of temperatures from the rectum and esophagus, (c) measurement of temperature from the surface of the tympanic membrane, and (d) measurement of temperature from middle ear cavity. Approval (REF: 2012/SHS/712) from SingHealth IACUC was obtained for the purpose of conducting Experiments 2 and 3 involving a live adult primate. The experiments were conducted in SingHealth Experimental Medicine Center animal facility that conformed to the National Advisory Committee for Laboratory Animal Research (NACLAR) guidelines.

Throughout each experiment runtime, the primate was sedated with inhaled Isoflurane anesthetic agent (general anesthesia) and its vital signs were closely monitored by a veterinarian. The primate was provided with appropriate medical treatments and adequate nutrition. Sufficiently long rest duration was also enforced on the primate in-order to allow it to rest and recuperate before being subjected to another round of experiment of the similar nature.

## Results and discussions

The results from the experiments are interpreted and analyzed according to the two focal points of the paper.

### Temperature uniformity in the tympanic membrane vicinity and measurement point selection

The ambient temperature *T*_*am*_, *T*_*tm*,*A*_ at point A and *T*_*tm*,*B*_ at point B of the tympanic membrane, as well as the ear canal temperature *T*_*cn*_ at Point C were measured as 22.5, 37.0, 36.4 and 36.1°*C* respectively at steady state. As suggested by Sato *et al* [22], the spot with the higher temperature is the preferred spot since this point is likely to be the least sensitive to ambient disturbances. An interesting observation from the results is that Point B registered a lower temperature than Point A, and Point B is within the lower anterior quadrant of the tympanic membrane suggested by Brinnel and Cabanac [21] to capture a temperature measurement. These results show that arbitrary point selected in this region may not necessarily return a highest temperature compared to other points on the tympanic membrane surface. If such a point does exist in this quadrant, it may require a search over the zone to pick up the highest temperature point. On the other hand, Point A which is at the malleus bone can be more easily located to focus the probe at. This is also intuitive since Point A is at the part of the tympanic membrane which is thicker compared to the lower anterior quadrant and that may translate into a higher thermal resistance against ambient disturbances, as the FEA also showed.

The stability of *T*_*tm*,*A*_ was additionally studied for resilience against temperature rise at the skin from peripheral vasodilation. The subject did a very short exercise of push-ups and sit-ups lasting at a leisurely pace over not more than 5 minutes, to induce metabolism to increase at the legs, hands and stomach muscles. Over this short duration, the body’s thermoregulatory system is expected to seek a balance between the maintenance of the body’s core temperature and the release of perspiration to cool the body. Thus, vasodilation will occur to cause an increase in blood flow to transport excess heat generated from affected muscle tissues to the skin. Measurement taken at the canal (Point C) registered a rise to 36.7°*C* while *T*_*tm*,*A*_ remained resilient at the same 37.0°*C*.

**Fig 8:**
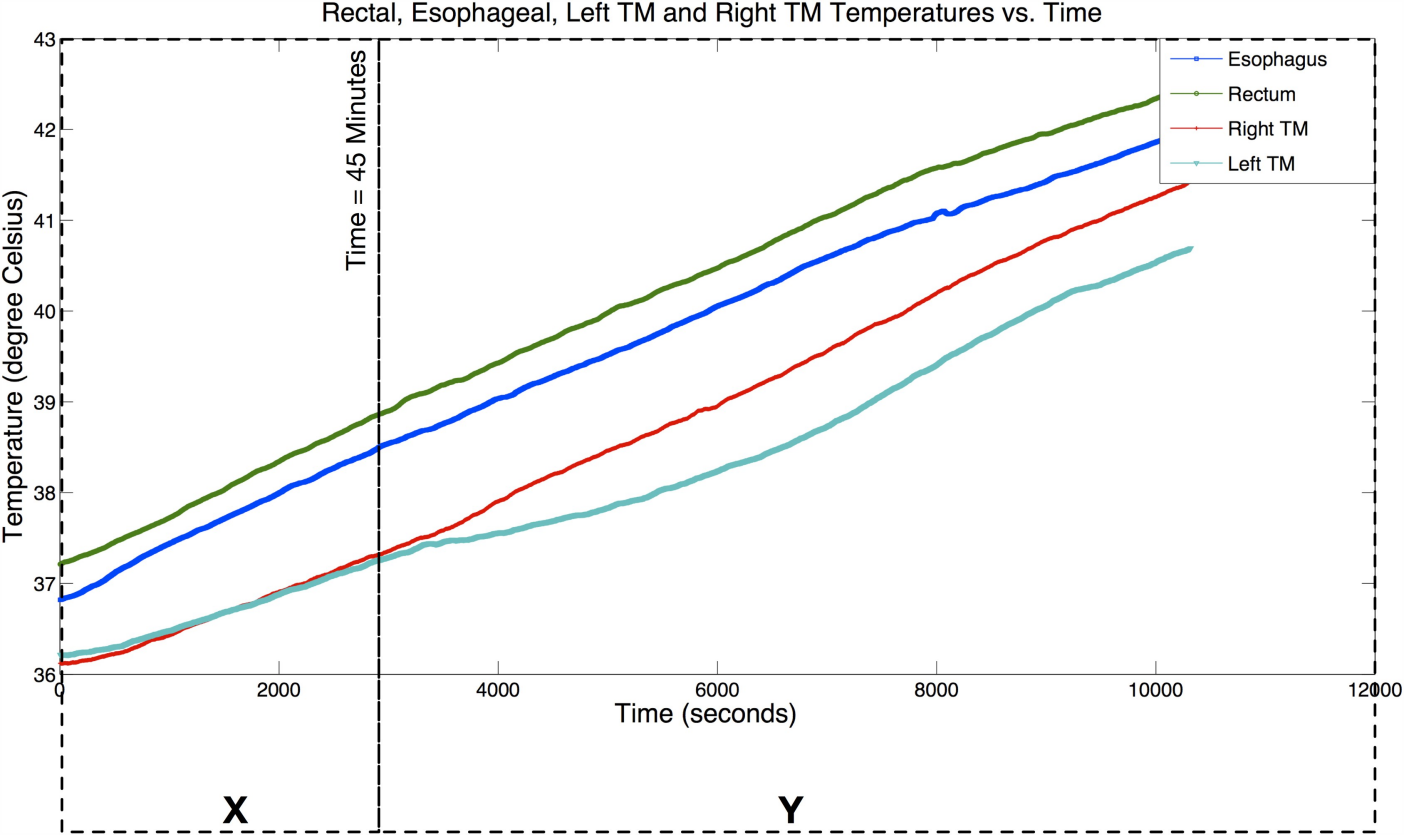
Temperature measurements results from Experiment 2. Primate body temperature measurements with Esophageal, Rectal and Tympanic Membrane (Left TM and Right TM) Temperatures plots. Experiment results are split into two test scenarios for Experiment 2: (1) *X*—Full Body Hyperthermia, and (2) *Y*—Full Body Hyperthermia with Left Face Fanning.

The sensitivity and effects of facial cooling on the temperatures from the various measurement sites can be observed from the results of Experiment 2. The initial temperature readouts from the temperature probes attached to the primate’s body were: (1) Rectum, *T*_*rm*_ = 37.2°*C*, (2) Esophagus, *T*_*es*_ = 36.8°*C*, (3) Left-Tympanic Membrane surface, *T*_*tm*,*L*_ = 36.2°*C*, and (4) Right-Tympanic Membrane surface *T*_*tm*,*R*_ = 36.1°*C*. 45 minutes into the experiment, convection skin cooling was introduced at the head area near to the left ear (left face fanning). Fig 8 shows the collected temperature readings at the four measurements points on the primate’s body. For clarity of view and interpretation, we split the plot into the two different test scenarios: (1) *X*—Full Body Passive Hyperthermia only, and (2) *Y*—Full Body Passive Hyperthermia with Left Face Fanning. *T*_*es*_ served as the reference temperature to compare against the *T*_*tm*,*L*_ and *T*_*tm*,*R*_.

The relationships between *T*_*tm*,*L*_ and *T*_*es*_, and between *T*_*tm*,*R*_ and *T*_*es*_ over the full time period of Experiment 2 (*X* + *Y* test scenario) can be explained by using the correlation and Bland-Altman analyses. The details of the analyses are furnished in Figs A1–A6 in S1 File. Correlation analyses indicate strong correlations between *T*_*es*_ and both *T*_*tm*,*L*_ (Pearson correlation coefficient (*r*) = 0.982, Sum of Squared Error (*SSE*) = 0.25, Probability in support of a null hypothesis (*p*)<0.001) and *T*_*tm*,*R*_ (*r* = 0.996, *SSE* = 0.15, *p* < 0.001). Bland-Altman analyses show the mean differences (*MD*) between these temperatures (*T*_*tm*,*L*_,*T*_*es*_) and (*T*_*tm*,*R*_,*T*_*es*_) are −1.408°*C* and −0.963°*C*, and the 1.96SD are −0.768°*C* and −0.606°*C*, respectively. The dynamic response of *T*_*es*_, *T*_*tm*,*L*_ and *T*_*tm*,*R*_ are stable and steadily increasing with chamber heating. Both *T*_*tm*,*L*_ and *T*_*tm*,*R*_ indicate very strong correlations with *T*_*es*_ with *r* → 1 even with left face fanning throughout the experiment though the correlation is higher on the right side. However, the respective *SSE* and *MD* vary with *SSE* = 0.25 and *MD* = 1.408°*C* for *T*_*tm*,*L*_, and *SSE* = 0.15 and *MD* = 0.963°*C* for *T*_*tm*,*R*_. Notably, there is a 40% in reduction of *SSE*, and 31.6% in reduction of |*MD*| for *T*_*tm*,*R*_, compared to *T*_*tm*,*L*_. Thus, the effects of face fanning on *T*_*tm*_ can be observed from this analysis.

The earlier analysis was done to compare *T*_*tm*_ in both ears with one side of the face undergoing fanning. It will be interesting too to examine the correlation on the side with fanning, before and after the onset of fanning. This analysis will show too if it is feasible to adopt a single CBT inference model for *T*_*tm*_ in the presence of such ambient interactions. To this end, we partitioned the experimental data in Fig 8 into two sets corresponding to the *X* and *Y* tests scenarios (i.e., at *t* = 45 minutes) and performed the similar correlation and Bland-Altman analyses. Correlation analyses demonstrate strong correlation between the *T*_*es*_ and both *T*_*tm*,*L*_ (*r* = 0.994, *SSE* = 0.033, *P* < 0.001) and *T*_*tm*,*R*_ (*r* = 0.995, *SSE* = 0.036, *P* < 0.001) for *X*, and *T*_*tm*,*L*_ (*r* = 0.98, *SSE* = 0.21, *P* < 0.001) and *T*_*tm*,*R*_ (*r* = 0.998, *SSE* = 0.078, *P* < 0.001) for *Y*. Bland-Altman analyses indicate values of *MD* and 1.96SD between the *T*_*es*_ and both *T*_*tm*,*L*_ (*MD* = −0.978°*C*, 1.96SD = −0.647°*C*), and *T*_*tm*,*R*_ (*MD* = −0.999°*C*, 1.96SD = −0.763°*C*) for *X*, and *T*_*tm*,*L*_ (*MD* = −1.56°*C*, 1.96SD = −1.141°*C*), and *T*_*tm*,*R*_ (*MD* = −0.95°*C*, 1.96SD = −0.562°*C*) for *Y*. The values of *SSE* and |*MD*| prior to the introduction of left face fanning (*X* test scenario) are relatively small at 0.033 and 0.978°*C* respectively for left ear, and at 0.036 and 0.999°*C* respectively for right ear, and both left and right ear have very similar values. With the introduction of fanning (*Y* test scenario), *SSE* and |*MD*| are 0.21 and 1.56°*C* respectively for left ear, and at 0.078 and 0.95°*C* respectively for right ear. We compare data for both ears during fanning and observe that *SSE* and |*MD*| are relatively higher for the left ear than the right ear. This issue highlights the difficulty of using a soft approach to infer the CBT solely from *T*_*tm*_ when it is affected by other factors such as environmental disturbances in this case. Measurements of these disturbances are necessary to serve as additional inputs to the model. To minimize these effects, a spot on the tympanic membrane which is resilient to these effects would be necessary.

### Middle ear cavity as CBT site

**Fig 9:**
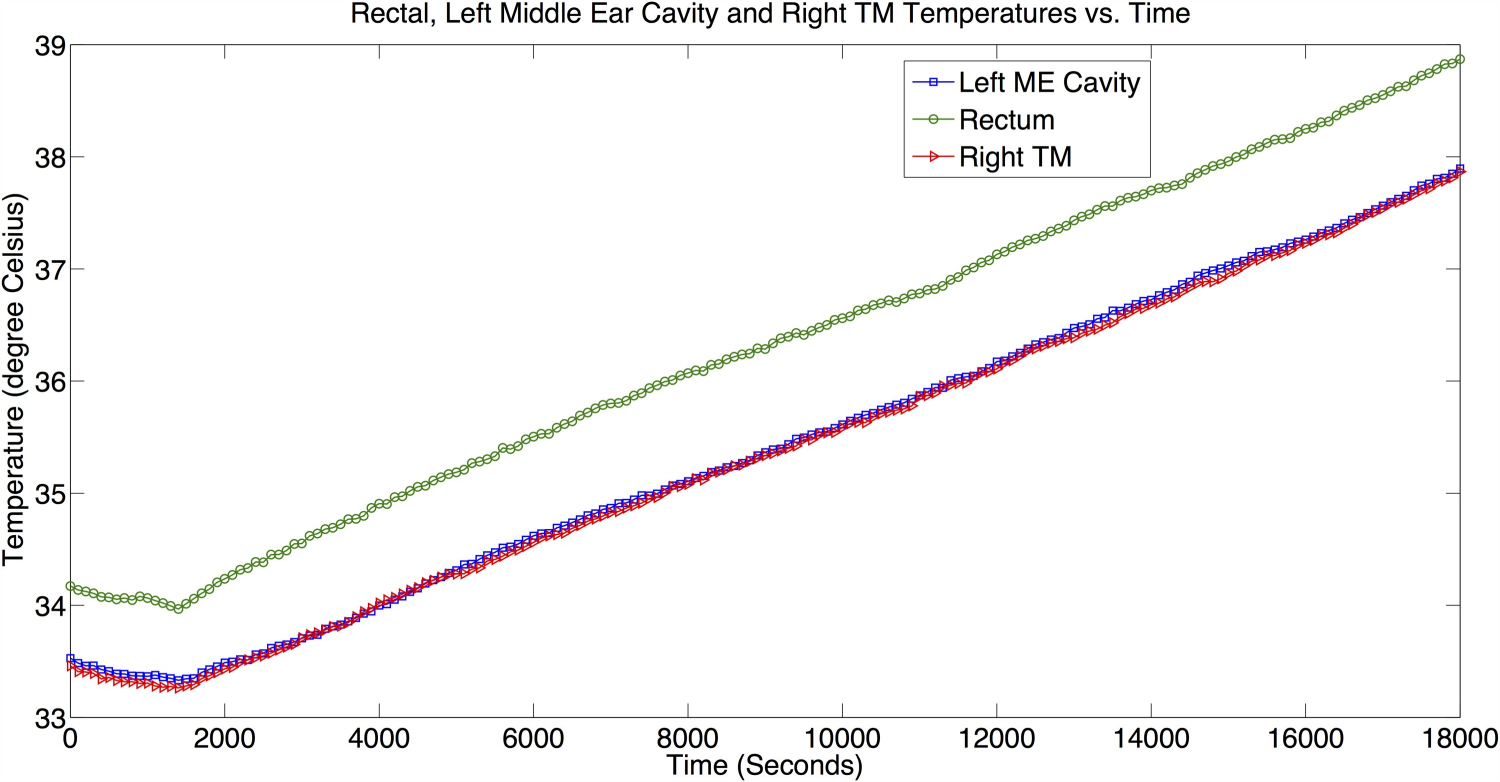
Temperature measurements results from Experiment 3*A*. *T*_*rm*_, *T*_*me*_ and *T*_*tm*,*R*_ vs. Time plots that depict behavior of each measurement site in Experiment 3*A* (No general anesthesia disruption).

Fig 9 reflects temperature measurements from Experiment 3*A* at the various sites: (1) Rectum, measuring *T*_*rm*_ (reference point), (2) Left middle ear cavity, measuring *T*_*me*_, and (3) Right tympanic membrane, measuring *T*_*tm*,*R*_.

Correlation analyses between *T*_*tm*,*R*_ and *T*_*rm*_, and between *T*_*me*_ and *T*_*rm*_ indicate very strong correlations (*r* → 1) between the *T*_*rm*_ and both the *T*_*tm*,*R*_ (*r* = 0.9997, *SSE* = 0.03, *p* < 0.001) and the *T*_*me*_ (*r* = 0.9997, *SSE* = 0.029, *p* < 0.001). Bland-Altman analyses show the MD between these temperatures (*T*_*tm*,*R*_ − *T*_*rm*_ and *T*_*me*_ − *T*_*rm*_) are −0.968°*C* and −0.931°*C*, and the 1.96SD are −0.855°*C* and −0.835°*C*, respectively. From the analyses, we observe for all the temperature pairs, their correlation coefficient *r* → 1, which suggest strong correlation for each temperature pair, indicating *T*_*me*_ and *T*_*tm*,*R*_ correlate very well with *T*_*rm*_. The details of the analyses are furnished in Figs B1–B2 in S1 File.

**Fig 10:**
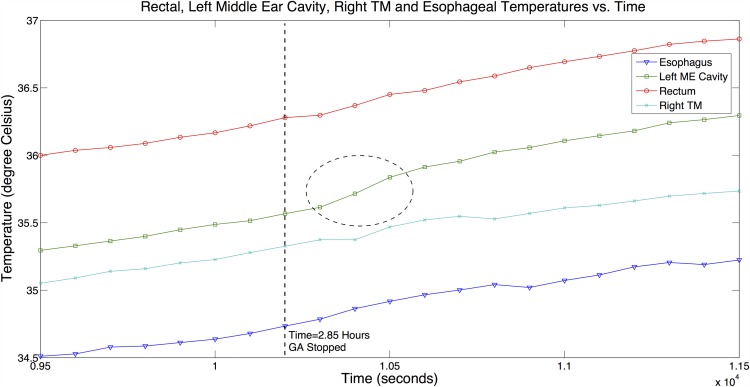
Temperature measurements results from Experiment 3*B*. *T*_*rm*_, *T*_*me*_, *T*_*tm*,*R*_ and *T*_*es*_ vs. Time plots that depict behavior of each measurement site with general anesthesia being disrupted in Experiment 3*B*.

In Experiment 3*B*, the effects of general anesthesia disruption and the corresponding change in CBT were observed from temperature measurements at various sites. The procedures of Experiment 3*A* were again carried out and in addition, a probe was inserted into the esophagus to measure *T*_*es*_. At 2 hours and 51 minutes into the experiment, the administration of general anesthesia was stopped for 15 minutes. Fig 10 shows the measurements at all the selected sites during the time window when general anesthesia was disrupted.

It is well-known that the administration of general anesthesia will lower CBT [1, 9]. This was observed at the start of all experiments when general anesthesia was administered. Temperature measurements at all sites in steady state were well below the healthy temperature range. After general anesthesia was halted in Experiment 3*B*, we observed from Fig 10, a clear and steeper change in the gradient of *T*_*me*_ (marked with a circle in the figure) showing an increase in the rate of change of *T*_*me*_. This observation is consistent with the presumption that the primate’s body thermoregulation function was gradually being restored with the removal of general anesthesia. This phenomenon is only observed in the *T*_*me*_ measurements and not evident from a visual inspection of other measurements over the same duration.

We split the data in Fig 10 into two measurement groups separated at *t* = 2.85 hours (2 hours and 51 minutes) boundary: (1) *G*_1_—Primate was administered with general anesthesia in a continuous and controlled manner, and (2) *G*_2_—General anesthesia administration to the primate was halted for 15 minutes.

The data in *G*_1_ group was first analyzed. Correlation analysis demonstrates strong correlation between *T*_*es*_ and *T*_*me*_ (*r* = 0.995, *SSE* = 0.009, *p* < 0.001). Bland-Altman analysis shows the mean difference between temperatures *T*_*es*_ and *T*_*me*_ is +0.812°*C*, and the 1.96SD is +0.851°*C*. The data in *G*_2_ group was next analyzed. Correlation analysis still demonstrates a strong correlation between *T*_*es*_ and *T*_*me*_ (*r* = 0.971, *SSE* = 0.02, *p* < 0.001). Bland-Altman analysis shows the mean difference between temperatures *T*_*es*_ and *T*_*me*_ is +0.852°*C*, and the 1.96SD is +0.915°*C*.

For comparison purpose, similar analyses were conducted for (*T*_*es*_, *T*_*tm*,*R*_) for both *G*_1_ and *G*_2_ data groups. The details of the conducted analyses are furnished in Figs B3–B6 in S1 File. A summary of the analyses results are shown in Table 1 with consistent *p* < 0.001 for all. We observe from the table, *r* ≥ 0.99, and *SSE* ≈ 0.01 for all the temperature pairs in the *G*_1_ group, showing they are very highly correlated for temperature data in the *G*_1_ group.

**Table 1:** Correlations and Bland-Altman analyses results for Experiment 3*B*.

	G1		G2	
	*T*_*es*_, *T*_*me*_	*T*_*es*_, *T*_*tm*,*R*_	*T*_*es*_, *T*_*me*_	*T*_*es*_, *T*_*tm*,*R*_
*r*	0.995	0.995	0.971	0.994
*SSE*	0.009	0.008	0.02	0.005
*MD*	+0.812	+0.571	+0.852	+0.569
1.96SD	+0.851	+0.596	+0.915	0.599

We observe a small drop in *r*, and increase in *SSE* values when we analyzed the data from *G*_2_ group for the correlation pairs of (*T*_*es*_, *T*_*me*_). Other correlation pairs consistently maintain *r* ≥ 0.99 and *SSE* ≈ 0.01 in both *G*_1_ and *G*_2_ groups. Thus, there is a deviation in *T*_*me*_ measurements following the general anesthesia change which is not seen in the other temperature measurements.

In addition, from the results of Experiments 3*A* and 3*B*, we compare the differences between *T*_*tm*,*R*_ and *T*_*me*_ over two separate tests. Measurements of *T*_*tm*,*R*_ were taken at two different points. We observed from Experiment 3*A*, the *T*_*me*_ and *T*_*tm*,*R*_ measurements were closely identical and the temperature measurements from Experiment 3*B* showed a notably greater difference between them with *T*_*me*_ being the higher measurement in both cases (Fig 10). In Experiments 3*A* and 3*B*, the temperature measurement points at the right tympanic membrane were selected differently. The primate’s ear canal is very restrictive (much more so than the human) to the insertion of the probe. There is no luxury of space to visually position the probe. In Experiment 3*A*, the vet could feel the presence of the bone when he engaged the tympanic membrane with the probe, but in Experiment 3*B*, there was no ‘hard’ contact feel. Thus, two distinctly different points could be invariably used in the experiments. Hence, the same observation as in Experiment 1 can be made that the *T*_*tm*_ measurement is sensitive to the measurement point.

We also observed from Experiment 3*B*, *T*_*tm*,*R*_ measurement was higher that *T*_*es*_, but in Experiment 2, *T*_*tm*,*R*_ was consistently lower than *T*_*es*_. It has been reported that the *T*_*tm*_ is higher than *T*_*es*_ [11, 12, 21]. The differentials between *T*_*tm*,*R*_ and *T*_*rm*_ are rather similar in both experiments, but the differentials between *T*_*es*_ and *T*_*rm*_ are different. It has been noted in previous studies that *T*_*es*_ measurement is sensitive to the placement of the probe and that a lower temperature can be obtained if it is too high up in the esophagus [10, 11].

### Other observations

The results from these experiments suggested that *T*_*tm*_ can be used to infer CBT if the probe is directed at a point on the tympanic membrane where stable measurements can be obtained. Otherwise, the effects on the measurements (such as head cooling) should be considered and used to compensate the actual measurements. The latter may not be efficiently viable from a practical perspective.

The measurements from the middle ear cavity were more responsiveness to changes in CBT. Inferencing a faster dynamical variable *T*_*me*_ from a slower *T*_*tm*_ is not easily achieved as it may require the computation of higher derivatives of the slower measurements and this is not practically viable if there exists significant measurement noise. A calibrated relationship between the two variables in the steady state may be more realistic in a soft model. However, this would require a situation wherein temperatures can be allowed to settle down with no additional heat stress or sink occurring in the process, which may or may not be detectable. Dynamically inferencing the CBT from a more sluggish measurement is thus a challenge which has not been truly resolved. Direct measurements of temperature from the middle ear cavity may represent one approach when real-time and dynamic indications of the CBT is needed.

## Conclusions

In this paper, we revisited the tympanic membrane vicinity as a potential measurement site for core body temperature (CBT). Two issues were explicitly targeted and experiments were carried out to verify our hypotheses and previous observations. First, the non-uniformity of temperature on the tympanic membrane surface was shown using a probe positioned at a pre-defined proximity to the tympanic membrane at predefined points. A point in front of the malleus bone (A), a point in the lower anterior quadrant (B) and a point at the distal end of the ear canal (C) were selected for evaluation. Point A registered the highest steady state temperature and showed stability in indicating the CBT. Facial cooling has been verified to affect tympanic membrane temperatures, but this sensitivity can be reduced by directing the measurement probe at the desired point. Secondly, the middle ear cavity was explored for the first time as a CBT site. The rapid responsiveness of the middle ear temperature towards a change in the CBT was observed relative to other sites. From the measurements, it is generally difficult to infer this change in middle ear temperature from the temperatures measured at the other sites. The middle ear cavity appears to be a potential CBT site to explore when real-time continuous measurements of CBT are necessary.

## Supporting information

S1 File Correlation and Bland-Altman analyses for Experiments 2 and 3. The file contains Figs A1–A6 (Appendix A) that represent Analytical results for Experiment 2, and Figs B1–B6 (Appendix B) that represent Analytical results for Experiment 3.
(PDF)
