# Supplementary material for: Re-visiting the tympanic membrane vicinity as core body temperature measurement site
Source: PLoS One. 2017 Apr 17;12(4):e0174120. doi: 10.1371/journal.pone.0174120 (PMC5393563; doi:10.1371/journal.pone.0174120)
Supplement: S1 File — The file contains Figs A1–A6 (Appendix A) that represent Analytical results for Experiment 2, and Figs B1–B6 (Appendix B) that represent Analytical results for Experiment 3. (PDF) [file pone.0174120.s001.pdf]

Appendix A: Correlation and Bland-Altman Analyses for Experiment 2

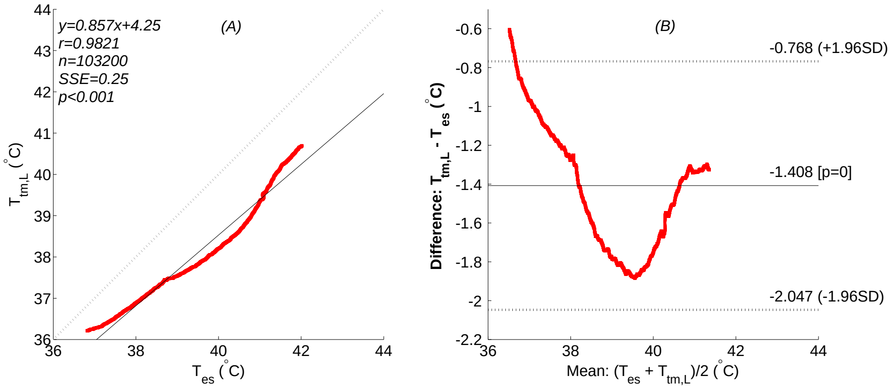

Figure A1. Analytical results for Experiment 2 that illustrate the effects of full body hyperthermia + full body hyperthermia with left face fanning ($X + Y$ test scenarios) on the left tympanic membrane temperature. (A) Relationship between left tympanic membrane temperature ($T_{tm,L}$) and esophageal temperature (T_{es}), and (B) the respective Bland-Altman plot.

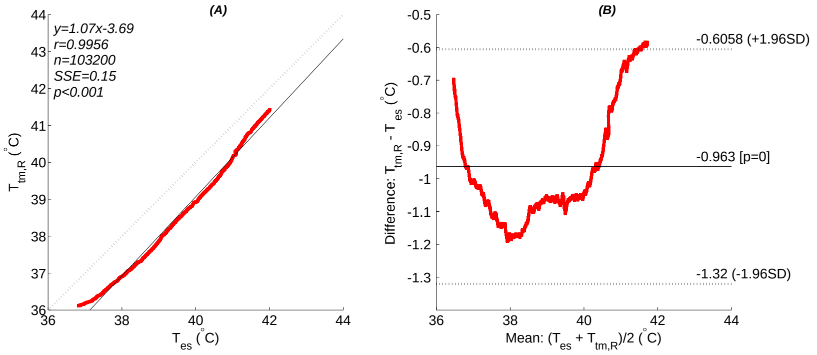

Figure A2. Analytical results for Experiment 2 that illustrate the effects of full body hyperthermia + full body hyperthermia with left face fanning ($X + Y$ test scenarios) on the right tympanic membrane temperature. (A) Relationship between right tympanic membrane temperature ($T_{tm,R}$) and esophageal temperature (T_{es}), and (B) the respective Bland-Altman plot.

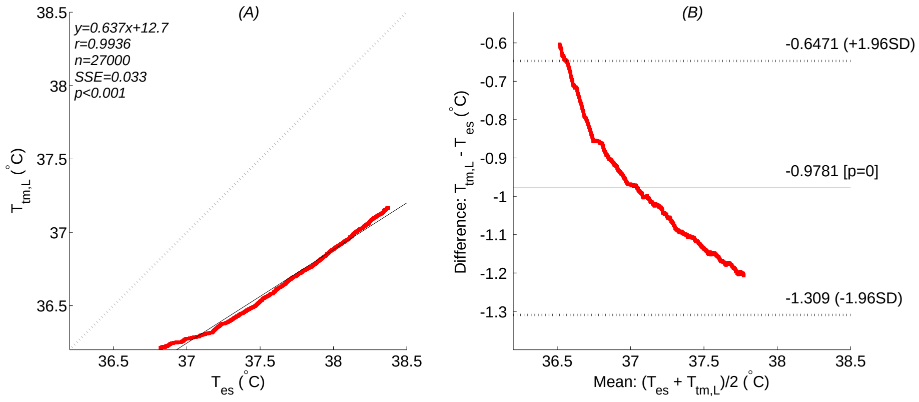

Figure A3. Analytical results for Experiment 2 that illustrate the effects of full body hyperthermia (X test scenario) on the left tympanic membrane temperature. (A) Relationship between left tympanic membrane temperature ($T_{tm,L}$) and esophageal temperature (T_{es}), and (B) the respective Bland-Altman plot.

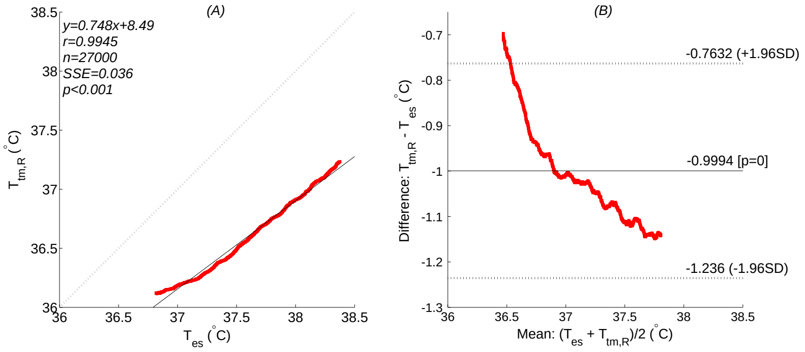

Figure A4. Analytical results for Experiment 2 that illustrate the effects of full body hyperthermia (X test scenario) on the right tympanic membrane temperature. (A) Relationship between right tympanic membrane temperature ($T_{tm,R}$) and esophageal temperature (T_{es}), and (B) the respective Bland-Altman plot.

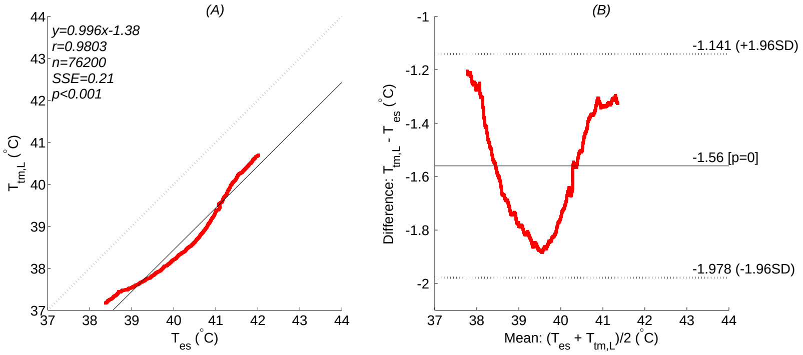

Figure A5. Analytical results for Experiment 2 that illustrate the effects of full body hyperthermia with left face fanning (Y test scenario) on the left tympanic membrane temperature. (A) Relationship between left tympanic membrane temperature ($T_{tm,L}$) and esophageal temperature (T_{es}), and (B) the respective Bland-Altman plot.

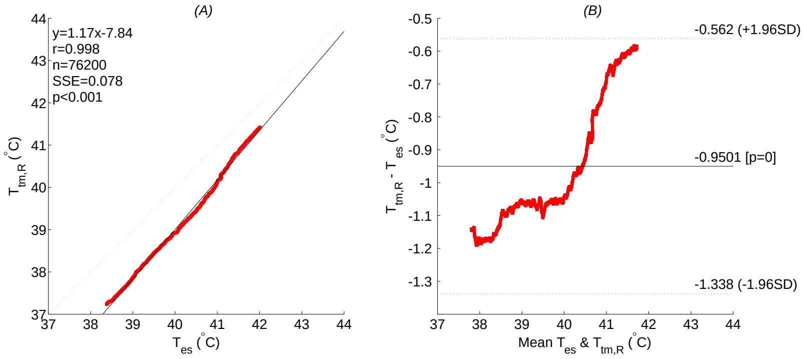

Figure A6. Analytical results for Experiment 2 that illustrate the effects of full body hyperthermia with left face fanning (Y test scenario) on the right tympanic membrane temperature. (A) Relationship between right tympanic membrane temperature ($T_{tm,R}$) and esophageal temperature (T_{es}), and (B) the respective Bland-Altman plot.

Appendix B: Correlation and Bland-Altman Analyses for Experiment 3

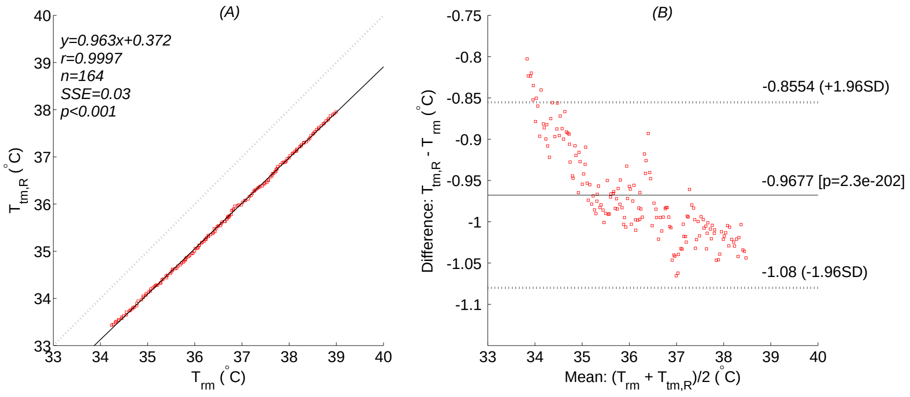

Figure B1. Analytical results for Experiment 3*A* that illustrate the effects of full body hyperthermia on the right tympanic membrane temperature. (A) Relationship between right tympanic membrane temperature ($T_{tm,R}$) and rectal temperature (T_{rm}), and (B) the respective Bland-Altman plot.

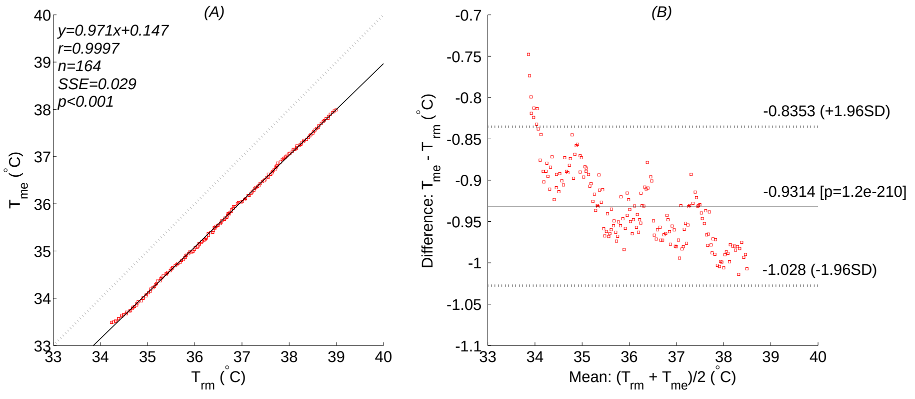

Figure B2. Analytical results for Experiment 3*A* that illustrate the effects of full body hyperthermia on the left middle ear cavity temperature. (A) Relationship between left middle ear cavity temperature (T_{me}) and rectal temperature (T_{rm}), and (B) the respective Bland-Altman plot.

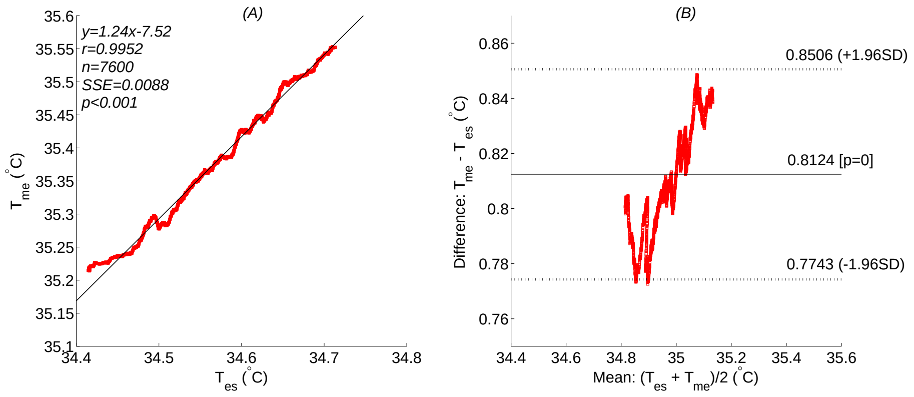

Figure B3. Analytical results for Experiment 3B that illustrate the effects of full body hyperthermia with GA on the left middle ear cavity temperature. (A) Relationship between left middle ear cavity temperature (T_{me}) and esophageal temperature (T_{es}), and (B) the respective Bland-Altman plot.

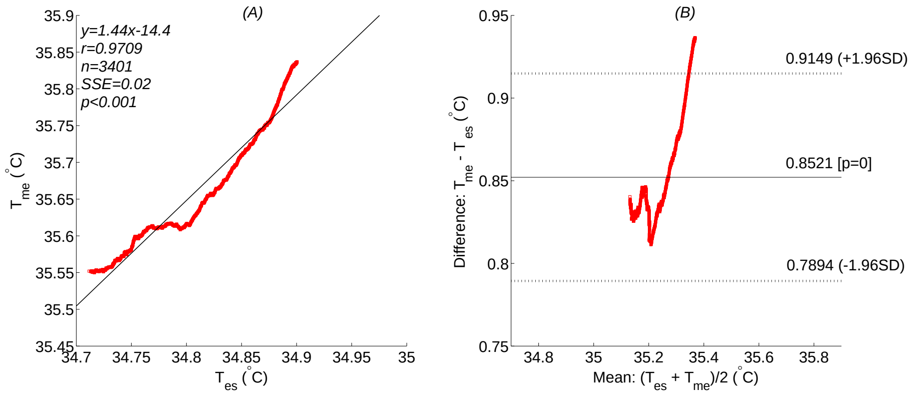

Figure B4. Analytical results for Experiment 3B that illustrate the effects of full body hyperthermia with GA halted (for 15 minutes) on the left middle ear cavity temperature. (A) Relationship between left middle ear cavity temperature (T_{me}) and esophageal temperature (T_{es}), and (B) the respective Bland-Altman plot.

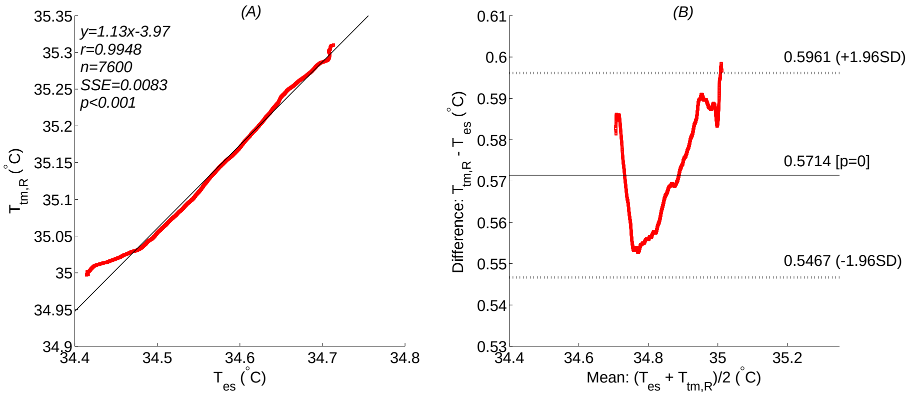

Figure B5. Analytical results for Experiment $3B$ that illustrate the effects of full body hyperthermia with GA on the right tympanic membrane temperature. (A) Relationship between right tympanic membrane temperature ($T_{tm,R}$) and esophageal temperature (T_{es}), and (B) the respective Bland-Altman plot.

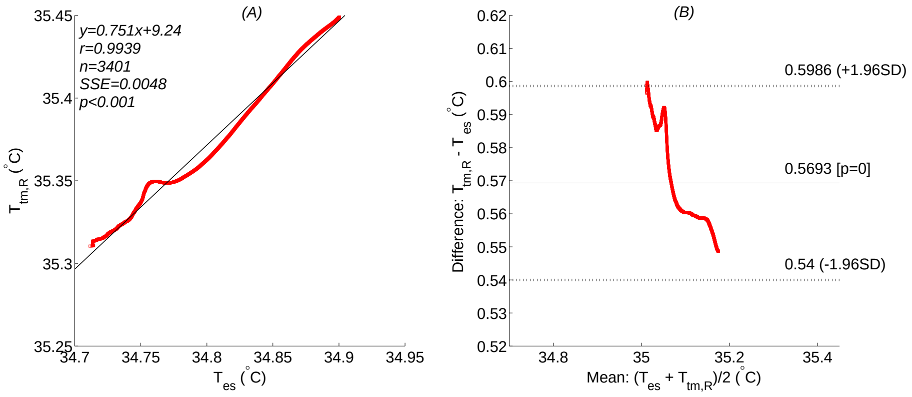

Figure B6. Analytical results for Experiment $3B$ that illustrate the effects of full body hyperthermia with GA halted (for 15 minutes) on the right tympanic membrane temperature. (A) Relationship between right tympanic membrane temperature ($T_{tm,R}$) and esophageal temperature (T_{es}), and (B) the respective Bland-Altman plot.
